# Microfluidics-assisted multiplexed biomarker detection for in situ mapping of immune cells in tumor sections

**DOI:** 10.1038/s41378-019-0104-z

**Published:** 2019-11-06

**Authors:** Daniel Migliozzi, Benjamin Pelz, Diego G. Dupouy, Anne-Laure Leblond, Alex Soltermann, Martin A. M. Gijs

**Affiliations:** 1 Laboratory of Microsystems, École Polytechnique Fédérale de Lausanne, 1015 Lausanne, CH Switzerland; 2 Lunaphore Technologies SA, EPFL Innovation Park, Building C, 1015 Lausanne, CH Switzerland; 3 Universitätsspital Zürich, Schmelzbergstrasse 12, 8091 Zürich, CH Switzerland

**Keywords:** Engineering, Microfluidics, Biosensors

## Abstract

Because of the close interaction between tumors and the immune system, immunotherapies are nowadays considered as the most promising treatment against cancer. In order to define the diagnosis and the subsequent therapy, crucial information about the immune cells at the tumor site is needed. Indeed, different types or activation status of cells may be indicative for specific and personalized treatments. Here, we present a quantitative method to identify ten different immuno-markers in the same tumor cut section, thereby saving precious samples and enabling correlative analysis on several cell families and their activation status in a tumor microenvironment context. We designed and fabricated a microfluidic chip with optimal thermomechanical and optical properties for fast delivery of reagents on tissue slides and for fully automatic imaging by integration with an optical microscope. The multiplexing capability of the system is enabled by an optimized cyclic immunofluorescence protocol, with which we demonstrated quantitative sequential immunostaining of up to ten biomarkers on the same tissue section. Furthermore, we developed high-quality image-processing algorithms to map each cell in the entire tissue. As proof-of-concept analyses, we identified coexpression and colocalization patterns of biomarkers to classify the immune cells and their activation status. Thanks to the quantitativeness and the automation of both the experimental and analytical methods, we believe that this multiplexing approach will meet the increasing clinical need of personalized diagnostics and therapy in cancer pathology.

## Sensors: Microfluidic detection of multiple biomarkers for cancer immunotherapy

Researchers in Switzerland have developed a chip to quickly and accurately identify different types of immune cells for cancer immunotherapy. A joint team from Ecole Polytechnique Fédérale de Lausanne and Lunaphore Technologies SA built on existing microfluidic technology to engineer a tissue processor combining microfluidic channels to deliver reagents, heating elements to control the reaction, and a viewing window above the reaction chamber. An optical microscope is used to detect fluorescently labeled immuno-markers, and the team developed image-processing algorithms to map the immune and cancer cells within the tissue samples. They tested the new system by detecting ten markers and analyzing coexpression and colocalization patterns in a lung cancer sample. Future work will increase the number of markers detected. This technology will enable clinicians to provide personalized diagnoses to cancer patients and adapt immunotherapy accordingly.

## Introduction

Evidence of a close interaction between tumors and the immune system has been reported for several typologies of cancer: gastrointestinal^1^, ovarian^2^, lung^3^, and pancreatic^4^ among others. Studies on the presence of immune cells in the tumor microenvironment suggest their strong interplay with the cancer cells^5–8^, and thus immunotherapies have been gaining much interest as effective treatment against cancer^3,9,10^. In current clinical evaluation of cancer severity, the activation status of the immune system in the surroundings of the tumor site is a key aspect to investigate^11–18^. To detect the presence of specific cell types, the standard practice is to perform straining on tissue slides from tumor biopsies by using marker-specific antibodies^19^ (Abs). From the detection of several cell types on the same tissue slide, oncologists can gain information on the potential interaction between them, which can be crucial to understand the interplay between the immune system and the cancer cells. Moreover, because of the highly invasive procedure to obtain tumor biopsies, staining for more than one biomarker on a same tissue slide enables to save such rare and precious samples.

Among the methods for multiplexed detection of markers on a same tissue slide, there are those, such as spectral deconvolution with confocal imaging^20^ and tyramide signal-amplification-based^21,22^, where the staining is performed for all the markers before the imaging step. The main drawback of these methods is the limited number of markers that can be detected simultaneously (<10 even by using multispectral imaging). To overcome this limit, methods such as staining with DNA-barcoded Abs^23,24^, microfluidic compartmentalization combined with quantum-dot labeling^25^, cyclic staining with fluorescently labeled Abs alternated with chemical inactivation^26^ or elution^27,28^ have been developed. The last two techniques also decrease the complexity of the assay by not needing chemically modified Abs. The limit of chemical inactivation-based techniques is that they require the primary Ab (that targets the marker to detect) to have a fluorescent tag, which prevents exploiting the signal-amplification benefit of using an indirect staining (i.e. untagged primary Ab + tagged secondary Ab). Furthermore, the unavailability of conjugated primary Abs for certain markers may prevent applicability or require an additional chemical-labeling step. Cyclic-staining techniques as described above allow the observation of >40 markers^26,28^. However, due to the need to perform many staining cycles most of these techniques are very time consuming and require intensive manual handling because the slides have to be mounted and unmounted in between staining cycles. Microfluidics have already been proved suitable to standardize fluorescent immunostaining on tissue sections^29–31^. For these reasons, we aimed at creating a microscope-integrated microfluidic platform to perform fast, quantitative and automated multiplexing and analysis of tissue sections for cancer immunology by using elution-based microfluidics-assisted cyclic immunofluorescence and image-based signal quantification and cell mapping. Important immune-cell families (and their corresponding biomarkers) are T-lymphocytes (CD3), cytotoxic T-lymphocytes (CD3, CD8), T helper lymphocytes (CD3, CD4), regulatory T-lymphocytes (CD3, FOXP3, CD4, CD25), B-lymphocytes (CD20), macrophages (CD68, CD11b), and natural-killer cells (CD56). Moreover, specific immune activation/inhibition can be assessed (PD-1 and PD-L1) as well as the development status (CD45RA for naïve and CD45RO for memory immune cells). Therefore, we developed a microfluidic multiplexing method for a selected subset of these markers (i.e. CD3, CD4, CD8, FOXP3, CD20, CD68, CD56, PD-1, and PD-L1), as well as a marker used to identify cells of epithelial origin (pan-Cytokeratins: CK), which highlights carcinoma cells. Among these markers, there are membrane, cytoplasmic and nuclear proteins, which can appear very differently when detected by optical imaging^24,26^. Therefore, to guarantee high-quality automatic detection for all biomarkers, we developed custom image-processing methods for each marker based on the morphology of the positive-stained cells.

## Results and discussion

### The glass-cyclic olefin copolymer microfluidic tissue processor and the integration with the optical microscope

**Fig. 1:**
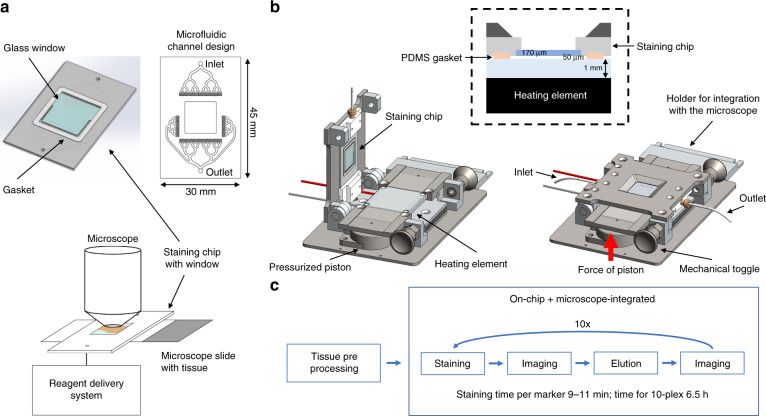
Look-Through Chip (LTC) and stainer for integration with an optical microscope. **a** COC chip with microfluidic channels, a PDMS gasket to create the reaction chamber on top of the tissue sample, and a glass coverslip to enable online imaging of the tissue slide. The exchange of reagents is done in a timeframe of 1 s, following the principle of the fast-fluidic exchange (FFEX) technology. **b** The LTC is mounted on the stainer and clamped to the glass slide with the tissue by two air-pressure-driven pistons which apply force vertically as indicated by the arrow. It also contains a heating element for precise control of the temperature of the chamber. Furthermore, the stainer can be mounted on a regular motorized microscope stage, thereby enabling the scanning of the tissue slide when clamped with the LTC. **c** Tissue preprocessing (including dewaxing and antigen retrieval) is performed off-chip. Subsequently, the slide is transferred to the stainer where staining, imaging and elution cycles are performed

The immunofluorescence stainings were performed using an adapted design of a microfluidic technology which has already been used to study cancer biomarkers on clinical samples^21,29,30,32–34^ and cancer cell lines^35,36^. The novel design of the microfluidic tissue processor reported here includes the integration of an optical-grade glass window in a cyclic olefin copolymer (COC) element: this enables direct microscopic observation of the tissue slide through the microfluidic chip during the experiment. Specifically, the present structure of the Look-Through Chip is composed of: a microstructured COC element that includes the microfluidic channels; an optical-grade glass coverslip that constitutes the imaging window; a gasket of polydimethylsiloxane (PDMS) that forms the reaction chamber (height of ≈50 µm) on the tissue slide. COC is a thermoplastics with attractive optical, chemical and thermal properties: chemical resistance to common solvents, high water barrier, low moisture uptake, and high heat deflection temperature^37,38^. Moreover, it is an inert material that is not contaminated by many chemicals used in clinical pathology protocols, and it is thermo-mechanically compatible with the fast heating/cooling of the sample for steps that require to operate at specific temperatures. Furthermore, the window made of an optical-grade glass coverslip (thickness of 170 μm) enables the use of high NA dry- or immersion-objectives, which require short working distance and high optical purity for suitable aberration correction. The design of the LTC with the microfluidic channels, gasket and glass window is depicted in Fig. 1a. The stainer (Fig. 1b) provides: the support for the clamping between the LTC and the tissue slide through pneumatic pistons; the temperature-conditioning system including a resistance thermometer, a Peltier element, and cooling-fins; the connection to the reagent delivery system; the support for the integration with the optical microscope. Such a system enables fast delivery of the reagents to the reaction chamber and precise temperature control: it takes less than 30 s to heat up from room temperature to 37 °C (for the staining steps) or from 37 to 50 °C (for the elution steps). Different from previous stainers^21,34^, the arrangement of microfluidic chip and microscope slide placement is inverted to enable direct optical access to the slide on an upright microscope through the LTC as shown in Fig. 1a. Mounting the stainer on a regular microscope stage of an upright microscope allows the movement of the whole assembly of LTC and tissue slide under the objective. This configuration enables fluorescent imaging by wide-field scanning through the imaging window (scanning area of 4 × 4 mm^2^) as well as performing fast staining cycles using the FFEX technology without needing to remove the sample from the microscope (Fig. 1c).

### Fluorescence signal identification and cell mapping

**Fig. 2:**
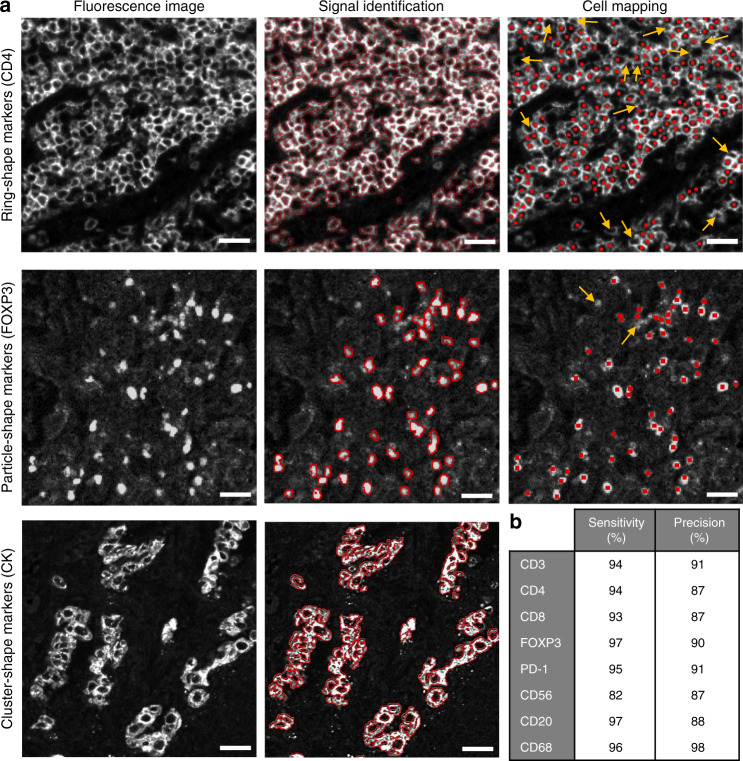
Image processing for signal identification and cell mapping. **a** Outcomes for ring-shape markers (CD4 in the image), particle-shape markers (FOXP3 in the image) and cluster-shape markers (CK in the image). Red outlines and dots indicate the border and the center of the cells automatically detected by the algorithm. Arrows indicate the cells that were missed by the algorithm Scale bars, 25 μm. **b** Quality-of-detection parameters resulting from the cell mapping

In order to evaluate the quality of the staining and to map each cell on the tissue, we created high-throughput computational algorithms that use the morphological properties of the fluorescence image of each biomarker to identify the signal pixels and the location of the cells. For ring-shape markers (CD3, CD4, CD8, CD56, CD20, and PD-1), we exploited the fixed thickness of the membrane staining to perform a local analysis around each pixel to enhance and threshold the local contrast of the image (see Methods for more details). This sharpening method enabled the selection of the signal pixel of the image, which was then confirmed by manual inspection by experienced pathologists. The subsequent step includes the computation of the ultimate eroded points of the signal image (see Methods for more details), which represent the approximate center of the cells (Fig. 2a). For particle-shape markers (FOXP3 and CD68), both steps are achievable with local contrast enhancement and thresholding combined with watershed segmentation (Fig. 2a). For cluster-shape markers (CK and PD-L1), for which the morphology is much less defined than for the other markers, the signal identification is based on attribute filtering and contrast thresholding, whereas the mapping is not possible in this case due to a lack of defined morphology of the cells (Fig. 2a). We evaluated the performance of the cell detection by manually assessing the detected cells (DC), the false positives (FP), the true positives (TP = DC − FP), and the false negatives (FN) (i.e. FP = cells wrongly detected as being positive, FN = cells wrongly detected as being negative) and by then evaluating the sensitivity (TP/[TP + FN]) and the precision (TP/[TP + FP]) of the image-based detection (Fig. 2b). The performance of the algorithms is very high, with sensitivity and precision of about 90%, which could represent a powerful means for oncologists when dealing with large sample areas containing millions of cells that would be impossible to analyze by direct visual inspection at the single cell level.

### Characterization of staining efficiency, elution efficiency and epitope stability

**Fig. 3:**
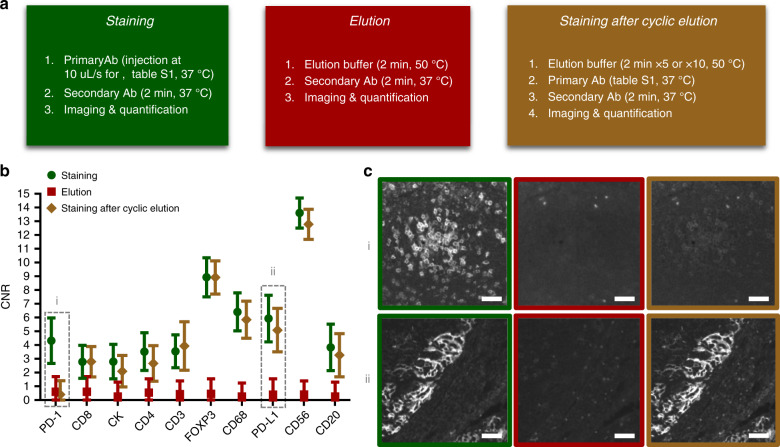
Protocol characterization for each biomarker. **a** Procedure for the assessment of the protocol steps. **b** Contrast-to-noise ratio (CNR) for the single biomarkers for staining, single elution cycle, and cyclic elution + staining. Data are plotted as mean ± SD. **c** Fluorescence images of the different steps for PD-1 (i) and PD-L1 (ii). Scale bars, 40 μm

Because of the advantages discussed previously, we used indirect immunofluorescent staining steps alternated with steps of elution. The staining conditions (Ab concentration and incubation time) are specific to each marker, but the overall duration of the incubations is 2–4 min for primary Abs and 2 min for secondary Abs, which guarantees fast and quantitative immunofluorescent staining, as described previously^29,35,36^. The elution step is the same for all the markers and consists in 2 min incubation in elution buffer at 50 °C. We assessed the staining and elution efficiency for all the markers of our panel independently on inflamed tonsil sections, where the presence of immune cells is guaranteed and can be used to robustly test each step of the protocol (Fig. 3a). To evaluate the ability to distinguish between signal and background, we used the contrast-to-noise ratio CNR = (*S* − *B*)/∆*B*, which is a parameter calculated from the average signal gray value (*S*), the average background gray value (B) and the standard deviation of the background gray value (Δ*B*). The CNR quantifies how high the signal is relatively to the background in terms of standard deviations of the latter, and thus represents the likelihood that the detected signal does not come from fluctuations of the background. For CNR > 2, more than two standard deviations separate signal and background, which guarantees high distinguishability. Moreover, since cells that are positive for a marker do not necessarily express the marker equally, the “true signal” may also vary, which means that also the CNR has a standard deviation that can be calculated (see Methods for more details). In Fig. 3b we report the CNR for each marker for several conditions. For the staining step (Fig. 3b, green dots), all the markers resulted in CNR > 2 for very short incubation time (Table S1). After the elution step (Fig. 3b, red squares), there is no signal remaining on the slide for any of the markers, thus resulting in low CNR. Importantly, the CNR reported here for the elution step is measured after re-staining with secondary Abs alone: this was done to demonstrate that the elution step not only removed the labeled secondary Abs, but also the unlabeled primary Abs. Finally, to evaluate the effect of the elution steps on the stability of the epitope, we performed a staining step (primary and secondary Abs) after several cycles of elution (five for PD-1, CD8, CK, CD4, CD3, and ten for FOXP3, CD68, PD-L1, CD56, and CD20). Figure 3b, c (brown diamonds) shows that detectability is preserved for all the markers except PD-1.

### Automated microfluidic multiplexing on tissue sections

**Fig. 4:**
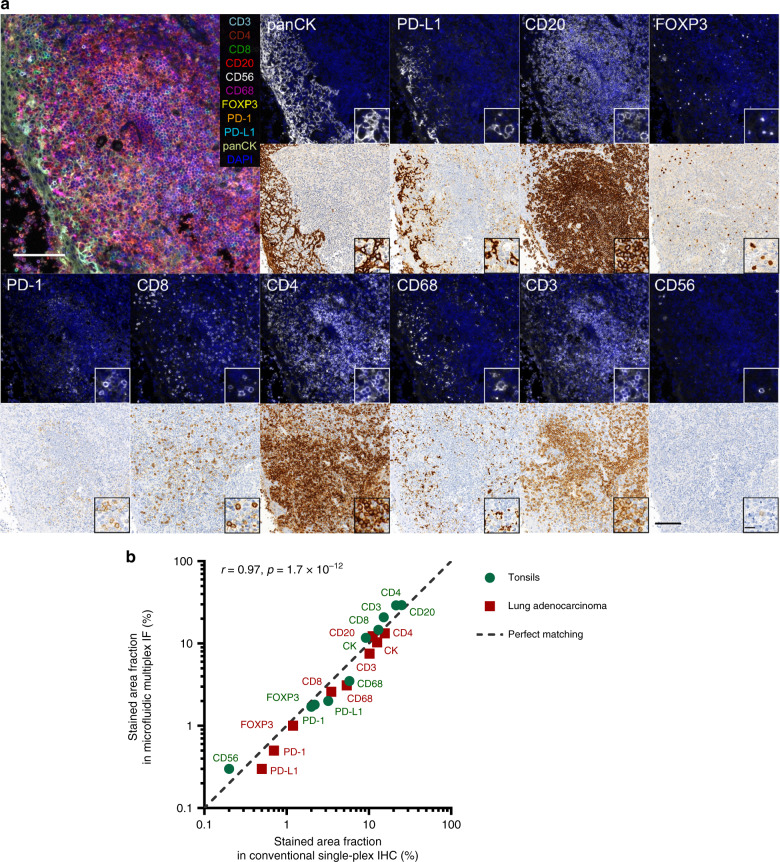
Automated microfluidics-assisted quantitative multiplexing. **a** Fluorescence images of biomarkers in tonsils from microfluidic 10-plex IF and bright-field images of conventional single-plex IHC on adjacent slides. Scale bars: 100 µm (overview images) and 15 µm (insets). **b** Comparison of the stained area fraction from microfluidic multiplex IF and manual single-plex IHC for each marker. The correlation coefficient *r* and its *p* value are reported in the same graph

Based on these single-biomarker results, we established that PD-1 should be stained at the beginning of the multiplexing protocol, because its staining signal decreases after elution. The other markers can be stained without loss of quality for all the elution cycles tested: five for CD8, CK, CD4, and CD3; ten for FOXP3, CD68, PD-L1, CD56 and CD20. Based on such considerations, we have chosen the following order: PD-1, CD8, CK, CD4, CD3, FOXP3, PD-L1, CD56, and CD20. This order guarantees that all the markers exhibit sufficient CNR to be detected at the right step of the multiplexing, and complete removal before staining the following marker. In a first step the autofluorescence of the tissue is recorded for the AF647 channel which is later used to subtract the autofluorescence from the images of the markers. The full staining of the individual markers including washing steps took 9−11 min, depending on the incubation time of the primary Ab. After each staining, the slide is imaged, subsequently undergoes the elution process and a final imaging step for control purposes. Compared to conventional immunostaining, where a single marker can take up to several hours to be stained and imaged, our method allows 10-plex staining including all imaging steps in less than 7 h. In Figs. 4 and S5, we compare the results of the microfluidic multiplex IF with conventional single-plex chromogenic immunohistochemistry (IHC), the current standard in clinical pathology. IF images with the corresponding IHC controls on adjacent tonsils (Fig. 4a) and lung cancer (Fig. S5) slices show that the biomarker patterns are conserved in the microfluidic elution-based multiplexing. CD3, CD4, CD8, CD20, CD56 and PD-1 show the ring-shape staining typical of markers widely expressed on the cell membrane. CD68 appears as puncta in cells positive for this marker. FOXP3, which stains a transcriptional regulator with nuclear localization, appears as a round particle. CK is a cytoskeleton protein and marks the cytoplasm of epithelial cells. PD-L1 is a transmembrane protein and can appear as clusters (Fig. 4a) or puncta (Fig. S4b). In order to quantitatively compare the two methods, we used the fraction of stained area (Fig. 4b). Concordance between conventional IHC and microfluidic multiplexed staining is observed for both tonsils and lung adenocarcinoma sections.

### Proof-of-concept analyses on coexpression and colocalization of biomarkers

**Fig. 5:**
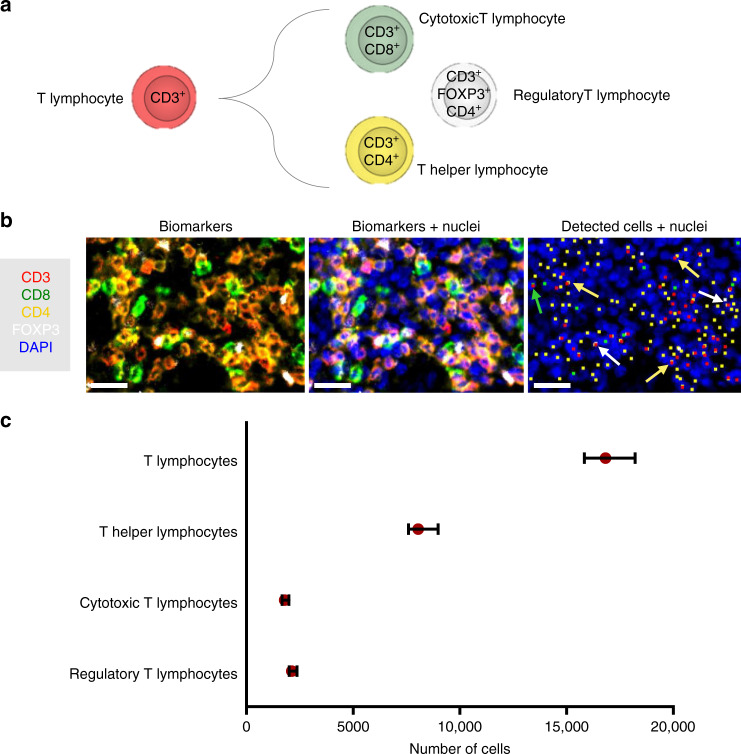
Proof-of-concept coexpression analysis on lung adenocarcinoma: T-cell phenotyping. **a** Schematics of T-cell differentiation with their expressed biomarkers. **b** Fluorescence images of biomarkers in a tissue section of lung adenocarcinoma. Colored dots in the third image are the detected cells for each biomarker. Colored arrows indicate cytotoxic (CD3^+^/CD8^+^, green), helper (CD3^+^/CD4^+^, yellow) and regulatory (CD3^+^/CD4^+^/FOXP3^+^) T lymphocytes. Scale bars, 30 μm. **c** Number of cells detected on the tissue section per each T lymphocyte type. Error bars indicate the predicted range for the true value of the number of cells, and are calculated based on sensitivity and precision reported in Fig. 1b

**Fig. 6:**
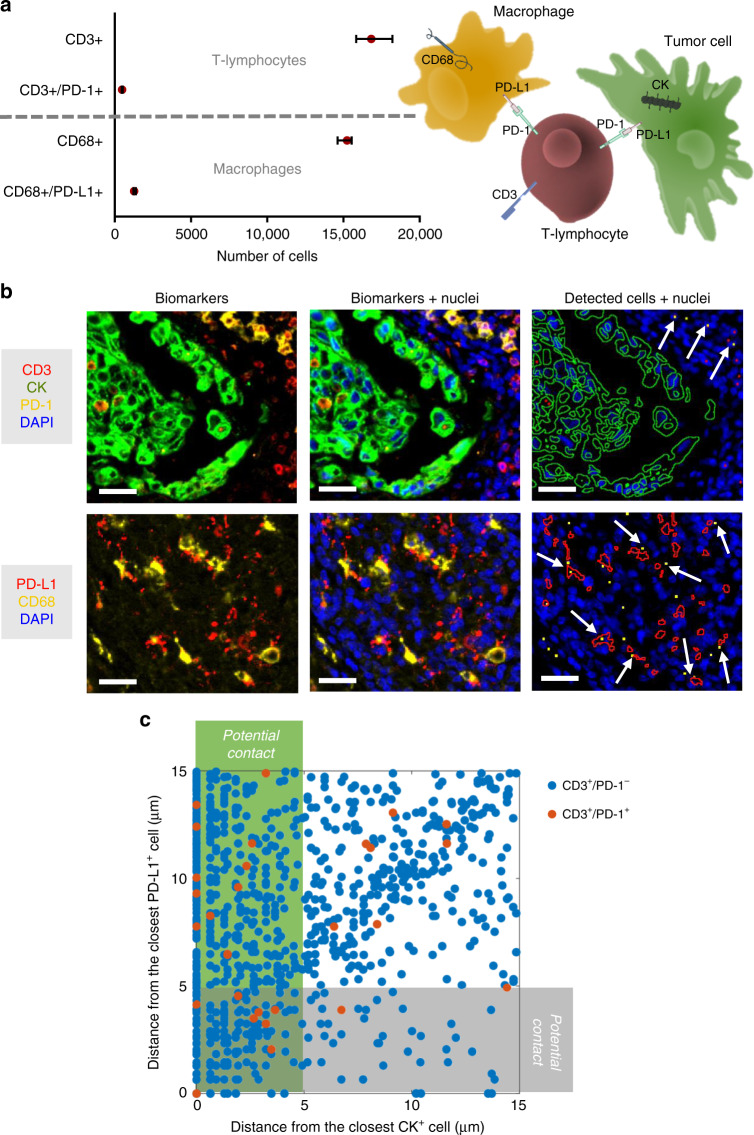
Proof-of-concept colocalization analysis on lung adenocarcinoma: immune response inhibition. **a** Number of cells expressing PD-1 and PD-L1 (left) and schematics of the cell types involved in the PD-1/PD-L1 inhibition pathway. Error bars indicate the predicted range for the true value of the number of cells, and are calculated based on sensitivity and precision reported in Fig. 1b. **b** Fluorescence images of biomarkers in a tissue section of lung adenocarcinoma. Colored dots and outlines in the third column are the detected cells and clusters for each biomarker. Arrows indicate PD-1 positive T cells (CD3^+^/PD-1^+^) and PD-L1-positive macrophages (CD68^+^/PD-L1^+^) in the top and bottom image respectively. Scale bars, 30 μm. **c** Colocalization analysis for T lymphocytes (CD3^+^). T cells lying within the green-shaded region may have anticancer action. PD-1^+^ T cells lying within the gray-shaded region may undergo immune inhibition

We took advantage of having all the markers on the same tissue slide to perform proof-of-concept coexpression and colocalization analysis on clinically relevant questions. As a first example, the importance of identifying the T lymphocyte subtypes (Fig. 5a) in the tumor microenvironment is crucial to efficiently address diagnosis and immunotherapy^2,4,14,16,18^. For this reason, we aimed at identifying the CD3+ cells (T lymphocytes) which also expressed CD4 (T helper lymphocytes), CD8 (cytotoxic T lymphocytes), or FOXP3 (regulatory T lymphocytes) in a lung cancer section (4 × 4 mm^2^). To perform this step, we used our cell mapping algorithm to identify the positive cells for each marker (Fig. 5b), and subsequently identified the double-positive cells by considering their proximity as detailed in the Methods-Data analysis section. In Fig. 5c we report the number of cells for each cell type. We observe that almost half (≈48%) of the T lymphocytes present in this section are T helper lymphocyte, but that also cytotoxic (≈11%) and regulatory (≈13%) T lymphocytes are present in the tumor microenvironment. Another fundamental aspect of T lymphocytes is their ability to be inhibited via specific signaling, such as the PD-1/PD-L1 pathway^10,39,40^: PD-1 is a membrane protein that can downregulate the immune system by suppressing T lymphocyte inflammatory activity when binding its ligand PD-L1, another membrane protein that can be expressed in cancer cells, macrophages and other cells. By using the same coexpression methodology as previously, we identified the PD-1 + T lymphocytes and the PD-L1 + macrophages in the same lung cancer case. Figure 6a reports the number of cells detected and Fig. 6b reports some clichés to illustrate colocalization of markers and cells. We also observed that only a minority of T lymphocytes express PD-1 in this lung tissue (≈3%), and similarly occurs for PD-L1 on macrophages (≈8%). As it was reported that proximity of immune cells to PD-L1+ cells may have an impact on PD-1-targeted therapy^41^, we calculated the center-to-center distance of each T-lymphocyte from the closest PD-L1+ and CK+ cells (Fig. 6c), to assess potential interaction between them. Given the accuracy of the cell-mapping algorithm for the localization of cells (see Methods for more details), CD3+ cells located closer than ≈5 μm to a CK+ cell are potentially in contact with it (green region in Fig. 6c). Similarly, PD-1+ T cells which are closer than ≈5 μm to a PD-L1+ cell have higher chance to be in contact with those cells (gray region in Fig. 6c). This way, one can estimate the likelihood of anticancer action or immune-cell inhibition, respectively. We can observe that in the imaged area (4 × 4 mm^2^), about 1/3 of the PD-1+ T cells (orange dots in Fig. 6c) may act as inhibitors for the immune reaction at the tumor site.

## Conclusion

The increasing need of multimarker detection and analysis in current clinical contexts makes it necessary to speed up and automate experimental and analytical techniques to help pathologists obtain quantitative information from precious samples such as tissue biopsies. We developed a microfluidic tissue processor that combines fast and quantitative immunofluorescent staining with the ability to image the tissue slide without the need to remove the microfluidic chip. The chip itself is composed of a microstructured polymeric part in COC, which has good mechanical and thermal properties that enable to rapidly vary the temperature during the protocol without harsh mechanical stress, while being inert to most chemicals used in such assays. A glass coverslip glued on the polymeric chip creates the ceiling of the microfluidic chamber and gives the optimal optical properties for fluorescence imaging, performed with a system that is fully integrated with an optical microscope. Overall, the microfluidic-based approach combined to the integration with the microscope for direct imaging after each step enables to perform a 10-plex staining in less than 7 h including all imaging steps. A comparable assay performed with traditional methods including mounting and unmounting for fluorescence imaging for each marker would require multiple days. Furthermore, we developed image-based cell mapping by using morphological properties of the different markers. The quality-of-detection parameters resulting from this procedure are very high (≈93% sensitivity and ≈90% precision for all the markers). Finally, coexpression and colocalization patterns were explored in proof-of-concept analyses on a lung cancer case. Further decrease of the experimental duration can be achieved by staining and imaging multiple markers in each cycle, e.g. by using a mouse and a rabbit primary antibody in combination with two secondary antibodies with different fluorescent labels. Also, the inclusion of the mapping for the cluster-shape markers can result from further analysis on their morphology. Due to sequential nature of the cyclic multiplexing, it would be easily possible to further increase the number of markers in the multiplex staining. This would allow better classification of specific cell lineages (for instance CD25 and CD11b would help in identifying regulatory T cells and macrophages, respectively) or the study of other immune checkpoint markers such as LAG-3 or TIM-3. Nonetheless, care has to be taken when adding new markers to the multiplexing panel, a full characterization including elution efficiency and epitope stability during elution should be performed. In fact, some epitopes may be particularly sensitive to the elution procedure^42,43^ (such as the one targeted by the anti-PD-1 Ab in this study): the low pH and the detergent may in fact affect the structure of the epitope, which will not be recognized by the Ab any more. This is an Ab-specific behavior, and previous studies reported this effect for epitopes of other markers^42^. However, this highly depends on the epitope itself and one should be careful in the choice of the Abs targeting particularly sensitive ones. Alternatively, different elution buffers or working temperatures may be explored. All in all, the microscope integrated cyclic immunofluorescence technique greatly facilitates the execution of high-plex stainings and thereby the discovery of novel tumor−microenvironment interactions.

## Methods

### Materials

Formalin-fixed paraffin-embedded (FFPE) slides for protocol characterization were purchased from East West Biopharma. They consisted of tonsils sections with chronic tonsillitis (4 μm thickness). FFPE slides for multiplexed detection were provided by the Institute of Pathology and Molecular Pathology, University Hospital Zurich, and consisted of 2-μm-thick sections of inflamed tonsils and lung adenocarcinoma. Tris buffered saline (TBS) 10× and Tween20 were purchased from Sigma (No. BP2471 and BP337-500). TBS 1× was obtained by diluting the concentrated TBS stock in deionized water. For immunostaining, the information about the antibodies are detailed in SI Table 1. Target Retrieval Solution (TRS) citrate pH 6, 10× was purchased from Dako (Code S169984). TRS was obtained by diluting the concentrated TRS stock in deionized water. Information about the primary Abs is found in the SI. Secondary Abs were purchased from Thermo Fisher Scientific: goat anti-mouse IgG labeled with Alexa Fluor Plus 647 (code A32728); goat anti-rabbit IgG labeled with Alexa Fluor Plus 647 (code A32733). Abs were diluted in TBS-0.05% Tween20 for the staining. The elution buffer consisted of IgG elution buffer pH 2.0 (Thermo Scientific, code 21028) and 1% sodium dodecyl sulfate solution (SDS) (Fisher Scientific, code BP1311-1). For reducing autofluorescence a 50 mM ammonium acetate buffer (Sigma-Aldrich, code 09691) with 10 mM CuSO_4_ (Sigma-Aldrich, code 1027841000) was used. Imaging buffer consisted of 1× TBS with 10 mM sodium ascorbate (Sigma Aldrich, A7631).

### Tissue slides preparation

Tissue slides were dewaxed twice with Histoclear II solution (National Diagnostics, USA) for 10 min and 30 s respectively. Then, they are rehydrated with a gradual ethanol series of 100, 100, 95, 70, 40%, for 10 s each before transferring the slides into tap water. Subsequently, antigen retrieval is performed at 95 °C for 40 min in Target Retrieval Solution 1×, and then stored in TBS 1× until staining. Alternatively, antigen retrieval was performed using a whole-polymer microfluidic chip (identical to the one of the multiplexing but with an opaque polymeric window) at temperatures over 100 °C for 10 min.

### Chip fabrication

The microfluidic channels were micro-milled in a 1-mm-thick COC sheet and a window of 8 × 8 mm^2^ was cut out in the center of the chip. #1.5 high precision glass coverslips (Carl-Roth, Germany) were cut to the right shape using a diamond tip pen and glued to the microfluidic chip by using UV-curable glue (NOA86, Norland, USA). The window allows to image a field of view of 4 × 4 mm^2^ of the tissue slide.

### Microfluidic setup

Glass slides with the manually preprocessed tissue sections were loaded into the stainer (Fig. 1b) which holds the look-through chip. The chip is interfaced with the slide via an elastomeric gasket forming a chamber of reaction of 17 × 17 mm^2^ with a height of ≈50 µm. The reagents are delivered into the chamber in a sequential manner as described previously^29^. The reagent delivery system consists of several reservoirs (50 and 2 mL volume) that can be used to deliver the reagents to the chip by pressure-driven flow. A graphical user interface on a computer allows the control of all steps including the imaging steps.

### Optical imaging

Images were acquired using an AxioImager M2m motorized epifluorescence microscope (Zeiss, Germany) with a ×20 0.8NA air objective. Zeiss filter cubes 02 and 50 were employed for DAPI and AF647 fluorophores, respectively, together with an X-Cite 120Q light source (Excelitas, USA). Slides were either mounted with a coverslip and Prolong Gold antifade mountant (Thermo Fisher, USA) or imaged directly through the LTC mounted on the stainer. If the imaging is performed directly through the LTC, the chamber was filled with imaging buffer to prevent the formation of radicals. Exposure times were set depending on the staining intensity and ranged between 30 and 500 ms. For the automated staining and imaging, the microscope was controlled using MicroManager 1.4^44^ and the image acquisition was started through the Matlab API. After scanning, tiles were stitched using Axiovision or FIJI software^45^. Brightfield images were acquired with a 3DHistech Pannoramic Midi II slide scanner using a Zeiss ×20 0.8NA objective.

### Image processing

Image processing is performed with FIJI. Independently from the morphology of the marker, the autofluorescence image (taken before any staining) is subtracted from the fluorescence image of each marker, in order to remove the nonspecific signal of the tissue. Then, a specific procedure is applied depending on the type of marker.

For ring-shape markers, the following steps are performed: a filtered version of the fluorescence image (Gaussian filter with 2-pixel radius) is subtracted to the raw image to enhance the local contrast; the local contrast (local maximum – local minimum) is calculated for each pixel by defining its neighborhood as a disk of 3 pixel radius; the pixels having both their local contrast higher than the defined threshold and their raw value higher than the defined mid-gray threshold are classified as SIGNAL; the complementary of the SIGNAL is classified as BACKGROUND after a 1-pixel erosion step, to avoid potential misclassification of pixels at the boundary between SIGNAL and BACKGROUND; the SIGNAL mask is then inverted and the ultimate eroded points (UEPs) calculated; the UEPs with a value lower than 5 are classified as detected cells and a CELL mask is created.

For particle-shape markers, the following steps are performed: a morphological closing (Disk filter with 2-pixel radius) is applied to the fluorescence image; a morphological white-top-hat filter (Disk filter with 15-pixel radius) is applied to the resulting image; the pixels having their value higher than the defined threshold are classified as SIGNAL; the complementary of the SIGNAL is classified as BACKGROUND after a 1-pixel erosion step, to avoid potential misclassification of pixels at the boundary between SIGNAL and BACKGROUND; the SIGNAL mask is segmented by binary watershed; all connected regions within a range of sizes (dependent on the marker) are classified as detected cells and a CELL mask is created.

For cluster-shape markers, the following steps are performed: a median filter is applied to the fluorescence image (Disk filter with 1-pixel radius); a gray-scale opening filter (30-pixel area) is applied to the resulting image; local Bernsen thresholding (radius = 5 pixels; contrast = 100) is applied to define the SIGNAL pixels; the complementary of the SIGNAL is classified as BACKGROUND after a 1-pixel erosion step, to avoid potential misclassification of pixels at the boundary between SIGNAL and BACKGROUND.

The signal and background quantification is performed on the raw images of the markers by using the SIGNAL and BACKGROUND marks.

The standard deviation of the contrast-to-noise ratio is calculated as the square root of the pooled variance of SIGNAL and BACKGROUND divided by the standard deviation of the background:

$$\Delta {\mathrm{CNR}} = \frac{{\sqrt {\left( {N_{\mathrm S}\Delta S^2 + N_{\mathrm B}\Delta B^2} \right)/\left( {N_{\mathrm S} + N_{\mathrm B}} \right)} }}{{\Delta B}},$$

where *N*_S_ and *N*_B_ are the number of SIGNAL and BACKGROUND pixels, respectively.

For IHC images, the following steps are performed: color deconvolution for hematoxylin and diaminobenzidine (DAB) staining is applied by using FIJI’s built-in color deconvolution tool with H DAB color vector; subsequently, the DAB image is inverted and thresholded to define the SIGNAL pixels; the complementary of the SIGNAL is classified as BACKGROUND after a 1-pixel erosion step, to avoid potential misclassification of pixels at the boundary between SIGNAL and BACKGROUND.

### Conventional immunohistochemistry and cyclic immunofluorescence on-chip

For chromogenic IHC, serial cuts were fully processed in the conventional Ventana BENCHMARK platform according to the manufacturer’s instructions. Information on the primary Abs are reported in the supplementary information. For the secondary Ab and detection via DAB, the Optiview DAB kit (#760–700, Ventana) was used. Sections were then counterstained with hematoxylin, covered with coverslip and scanned for imaging analysis.

For cyclic IF on-chip, the following steps were performed for each marker of the multiplexing panel: staining, imaging, elution and imaging (see Fig. 1c). The autofluorescence of the tissue was recorded before starting the first staining of the multiplexing. For the staining of an individual marker, mouse or rabbit primary antibodies were incubated for either 2 or 4 min (see SI). Fluorescently labeled secondary Abs were incubated for 2 min. Total duration of the staining of a single marker was between 9 and 11 min. Imaging was performed as described below. For the elution the tissue was incubated with elution buffer at 50 °C for 2 min before being washed with TBS. A second round of imaging was performed as control for elution performance (see supplementary information). The tissue was incubated with 10 mM CuSO4 in 50 mM ammonium acetate for 15 s after each elution step to reduce the autofluorescence of erythrocytes^46^. After the staining-imaging-elution-imaging cycle was finished for one marker, the same cycle was repeated for the next marker until all markers were stained and imaged.

### Data analysis

Coexpression and colocalization analyses are performed with FIJI and MATLAB^47^. To determine coexpression of two markers in the same cell, we select all the cells which are positive for the two markers independently and we selected the double-positive ones based on the precision of the calculation of the cell location during the cell mapping step: as the cell center is located with 3–4 pixels precision (≈ 2–2.5 μm) and the cell size is about 14–15 pixels (≈10 μm), centers of cells positive for two different markers and located less than 5 pixels apart (≈3 μm) are indeed the same cell, being positive for both markers. To perform this, the following steps are performed: the CELL masks of the two markers are enlarged by two pixels; all regions of overlap are classified as cells being positive for coexpression.

For the colocalization analysis, we created a mask for PD-L1 and CK by selecting all the pixels identified as signal in the corresponding fluorescence images. Then, we calculated the minimum distance between each T lymphocyte and the PD-L1+ and CK+ regions. Due to the same consideration as previously about the precision of cell localization, cells located at less than 5 μm from PD-L1+ or CK+ regions are potentially in contact with them.

## Supplementary information

Supplementary material
