# Supplementary material for: Microfluidics-assisted multiplexed biomarker detection for in situ mapping of immune cells in tumor sections
Source: Microsyst Nanoeng. 2019 Nov 6;5:59. doi: 10.1038/s41378-019-0104-z (PMC6831597; doi:10.1038/s41378-019-0104-z)
Supplement: Supplementary file 1 — Supplementary material [file 41378_2019_104_MOESM1_ESM.pdf]

Supplementary information

Antibodies

In table S1-2, we show the list of the antibodies used in the protocols, along with other related information.

Target	Host	Clone	Supplier	Dilution	Incubation time (min)	Exposure (ms)
CD20	Rabbit	SP32	Thermo Fisher Scientific	1:100	2	150
CD68	Mouse	KP1	Thermo Fisher Scientific	1:200	2	150
FOXP3	Rabbit	SP97	Thermo Fisher Scientific	1:25	4	300
PD-L1	Rabbit	E1L3N	Cell Signaling Technologies	1:100	4	200
CD56	Rabbit	MRQ-42	Cell Marque	1:400	2	200
CD3	Mouse	F7.2.38	Thermo Fisher Scientific	1:25	4	500
CD8	Rabbit	SP16	Thermo Fisher Scientific	1:50	4	400
CD4	Rabbit	EPR6855	Abcam	1:150	4	400
PD-1	Mouse	NAT105	Abcam	1:150	4	300
CK	Mouse	AE1/AE3	Thermo Fisher Scientific	1:100	4	200

Tab. S1 Information on primary antibody used in the microfluidic multiplexing.

Target	Host	Clone	Supplier	Dilution	Incubation time (min)
CD3	Mouse	LN10	Leica/Novocastra	1:500	32
CD4	Rabbit	SP35	Ventana-Roche	prediluted	32
CD8	Mouse	C8/144B	Dako	1:100	32
CD20	Mouse	L26	Ventana-Roche	prediluted	32
CD56	Mouse	123C3	Ventana/Roche	prediluted	32
CD68	Mouse	PG-M1	Dako	1:50	32
FOXP3	Mouse	236A/E7	Abcam	1:50	60
PD-1	Mouse	J116	eBioscience	1:50	32
PD-L1	Rabbit	E1L3N	Cell Signaling Technology	1:100	32
CK	Mouse	Lu5	BMA Biomedicals	1:250	32

Tab. S2 Information on primary antibody used in the conventional immunohistochemistry.

Image processing steps

In figures S1-2-3, we illustrate the procedure for the signal identification and the cell mapping as block diagrams. For the thresholding operations, we indicate the threshold values. The numerical values for the other operations are reported in the Methods section.

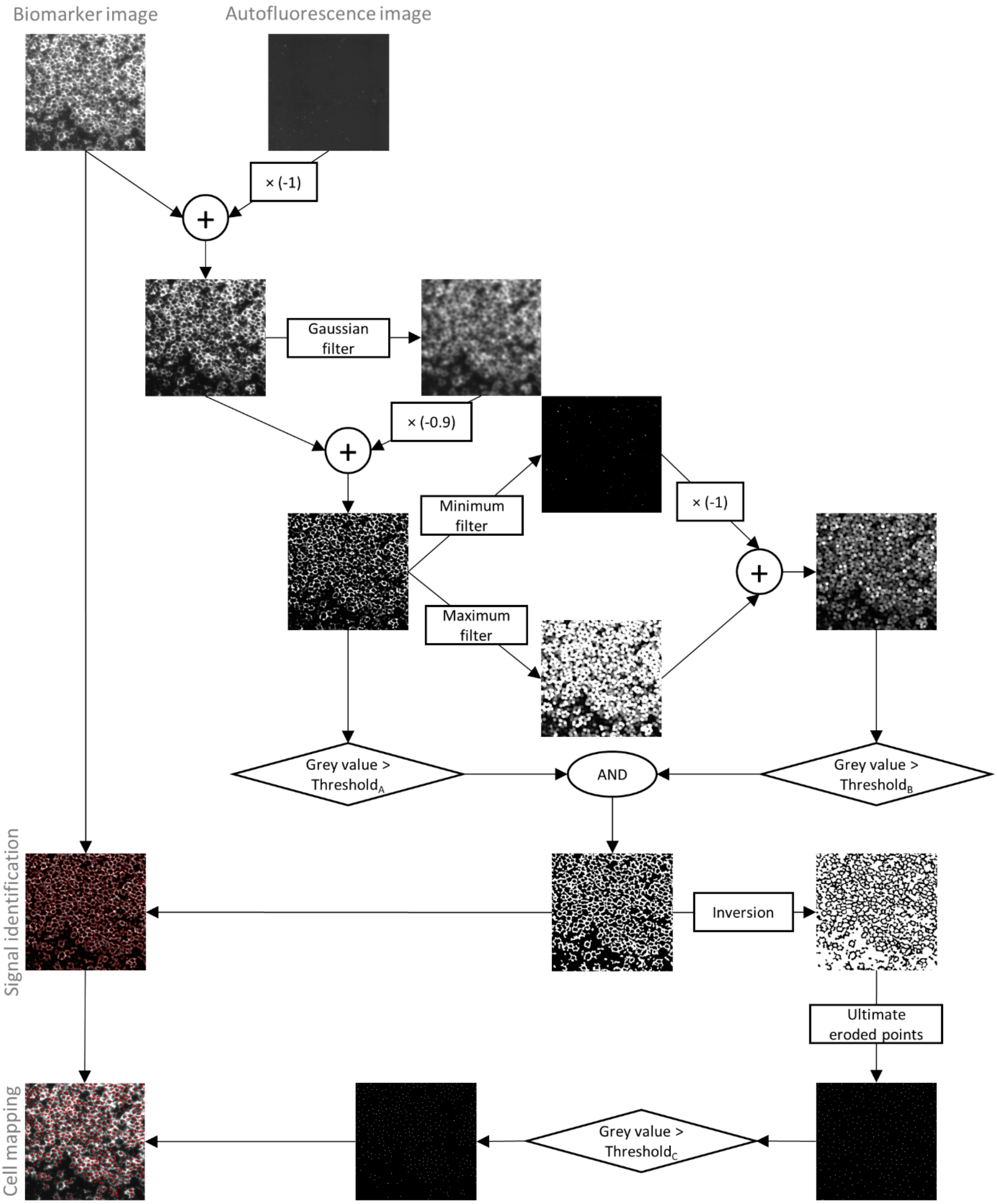

Fig. S1 Image-processing for ring-shape markers: Threshold_A = 0.2 × Local-Maximum + 0.8 × Local-Minimum; Threshold_B = marker dependent; Threshold_C = 5.

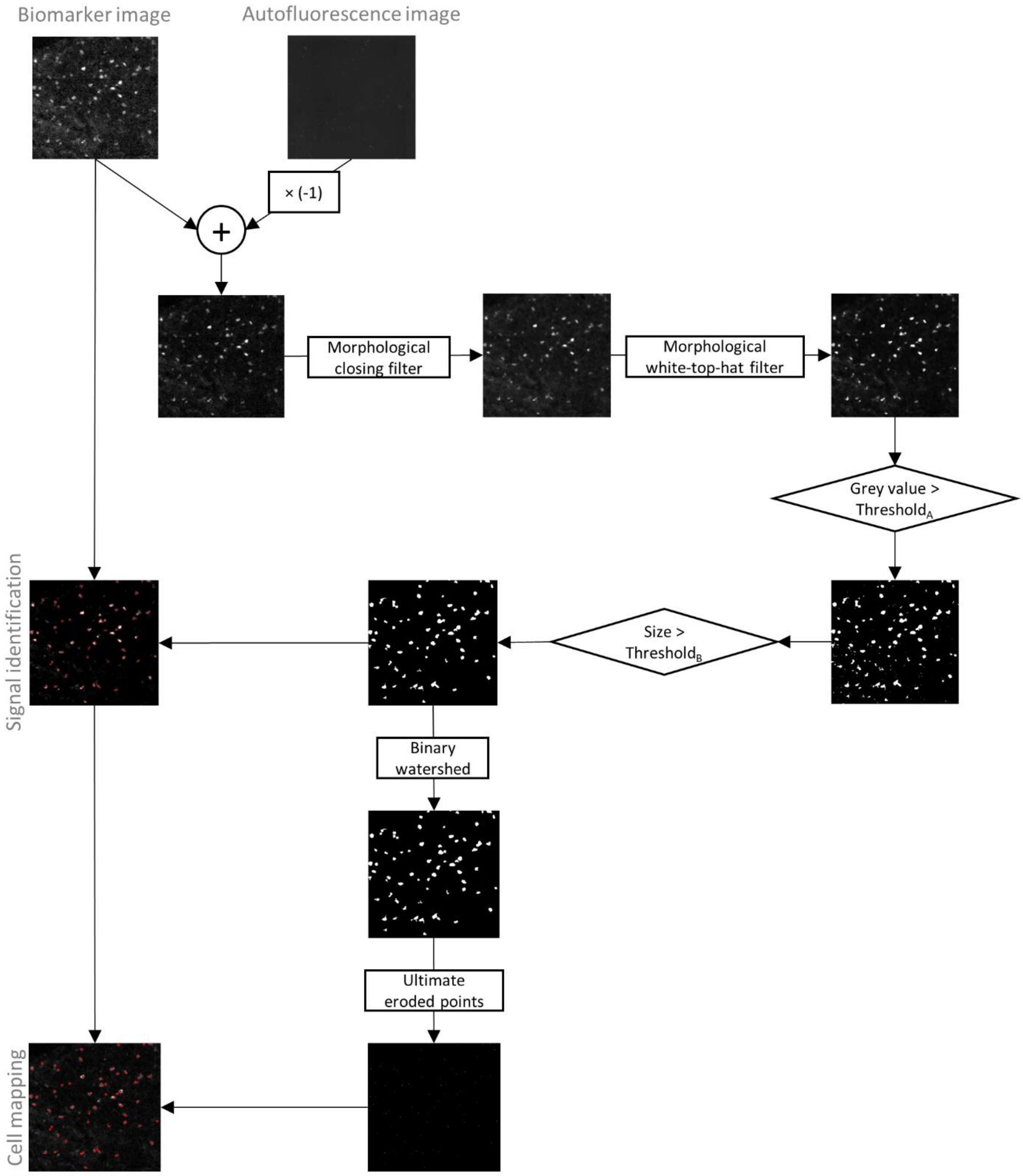

Fig. S2 Image-processing for particle-shape markers: Threshold$_A$ = marker dependent; Threshold$_B$ = 30.

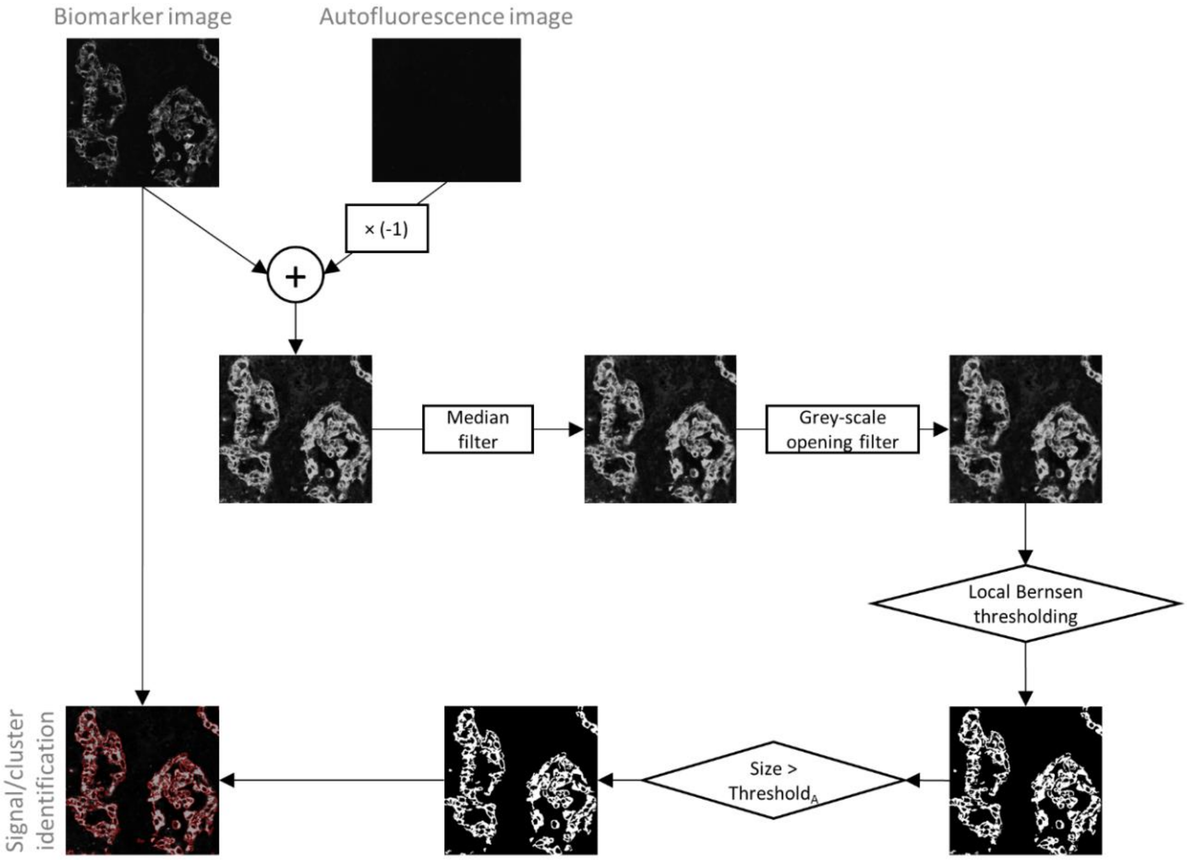

Fig. S3 Image-processing for cluster-shape markers: Local Bernsen thresholding (Radius=5 pixels, Contrast=100); $Threshold_A$=30.

Characterization of the detection parameters

In table S3, we report the number of cells analyzed to obtain the quality-of-detection parameters reported in figure 2b.

	CD3	CD4	CD8	FOXP3	PD1	CD56	CD20	CD68
**Detected Cells [DC]**	1125	1089	114	925	602	149	989	912
**False Positives [FP]**	102	138	15	97	53	20	116	15
**True Positives [TP=DC-FP]**	1023	951	99	828	549	129	873	897
**Falve Negatives [FN]**	63	59	8	28	31	29	31	38
**Sensitivity [TP/(TP+FN)]*100**	94	94	93	97	95	82	97	96
**Precision [TP/(TP+FP)]*100**	91	87	87	90	91	87	88	98

Tab. S3 Number of cells analyzed per marker to evaluate the quality of the detection.

Characterization of the protocol steps

In figure S4, we report (a) the normalized fluorescence intensity from which the CNR (fig. 3a) for the different steps were derived and (b) the fluorescence images of the different steps of the protocol.

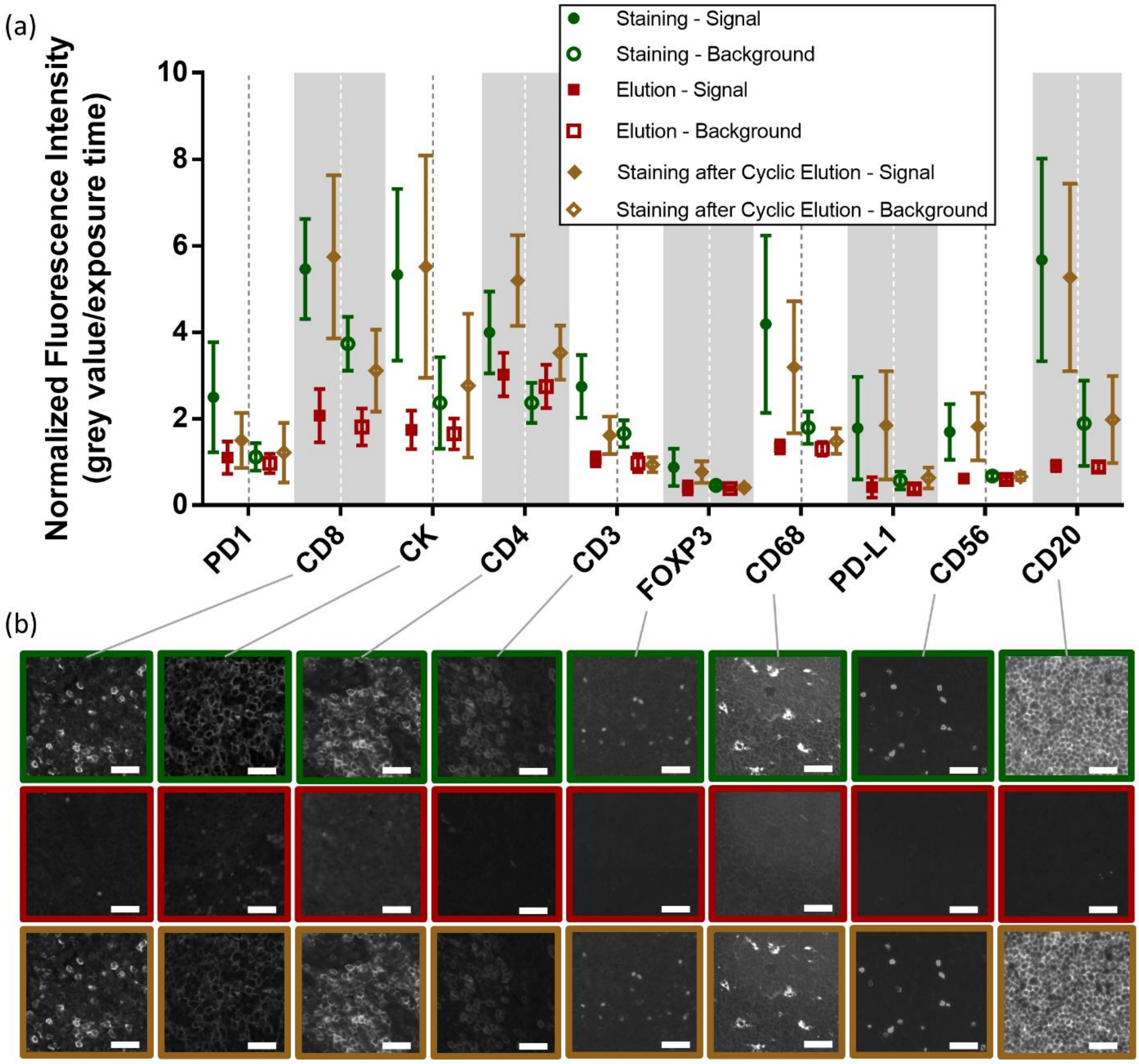

Fig. S4 (a) Characterization of the elution protocol. Normalized fluorescence intensity of the different steps of the characterization for both Signal and Background. Data are plotted as mean ± SD. (b) Fluorescence images of the different steps for the markers indicated. Scale bars, 40 µm.

IgG controls and microfluidic multiplexing on lung adenocarcinoma

In fig. S5-a, we report the control slides for both tonsils and lung adenocarcinoma stained with non-specific IgG Ab, which show that no structure is stained as expected. In fig. S5-b, we report a comparison between the microfluidic 10-plex IF and the conventional IHC for a case of ling adenocarcinoma.

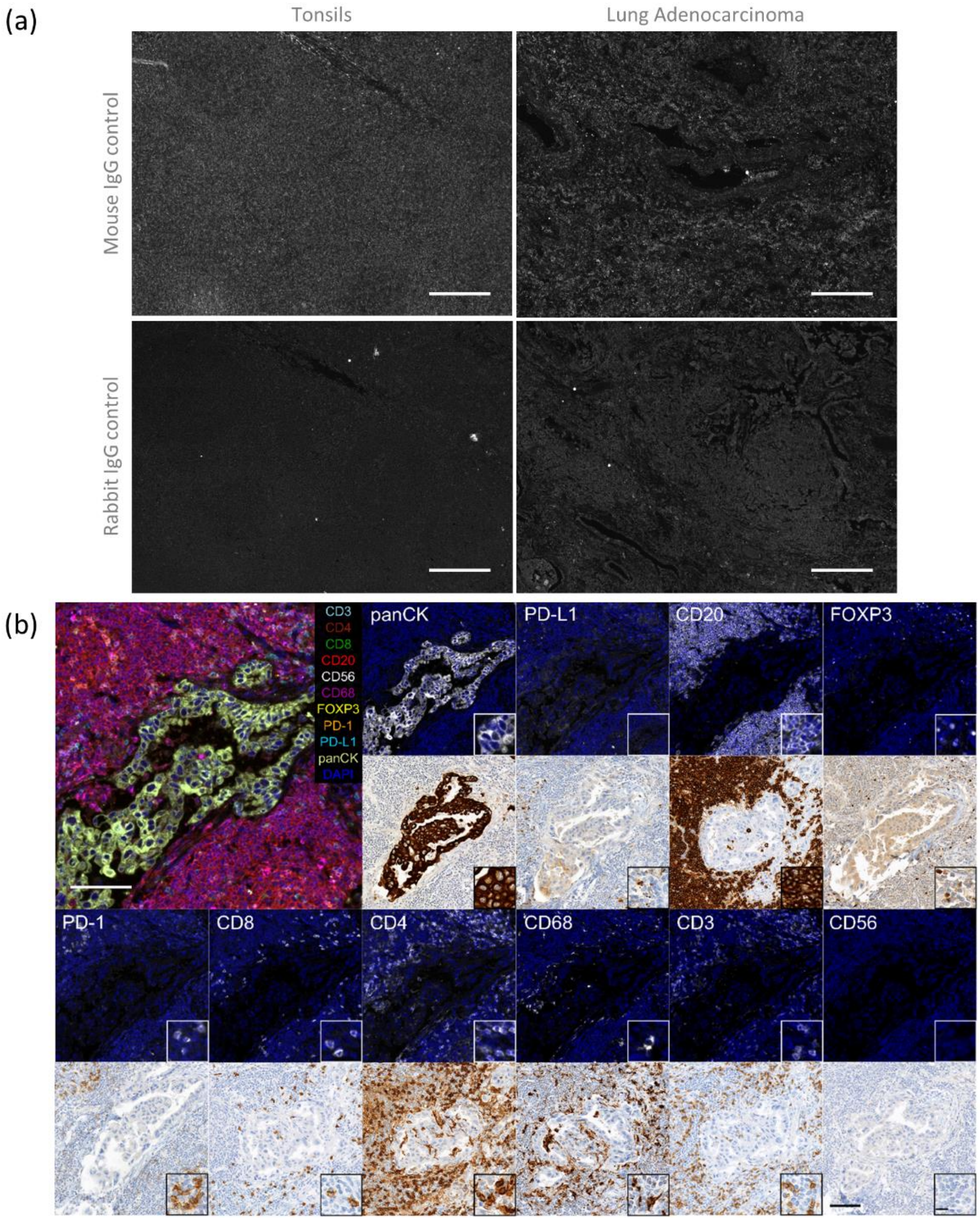

Fig. S5 IgG controls and microfluidic multiplexing on lung adenocarcinoma. (a) Fluorescence images of IgG controls tested with a concentration of 100 µg/mL for both primary IgG antibodies and the longest exposure (500 ms). (b) Fluorescence images of biomarkers in lung adenocarcinoma from microfluidic 10-plex IF and bright-field images of conventional single-plex IHC on adjacent slides. Scale bars: 100 µm (overview images) and 15 µm (insets).
